# A prognostic predictor panel with DNA methylation biomarkers for early-stage lung adenocarcinoma in Asian and Caucasian populations

**DOI:** 10.1186/s12929-016-0276-x

**Published:** 2016-08-02

**Authors:** I-Ying Kuo, Jayu Jen, Lien-Huei Hsu, Han-Shui Hsu, Wu-Wei Lai, Yi-Ching Wang

**Affiliations:** 1 Department of Basic Medical Sciences, College of Medicine, National Cheng Kung University, Tainan, Taiwan; 2 Institute of Clinical Medicine, College of Medicine, National Cheng Kung University, Tainan, Taiwan; 3 Department of Pulmonary Medicine, Chi Mei Medical Center, Tainan, Taiwan; 4 Division of Thoracic Surgery, Taipei Veterans General Hospital; Institute of Emergency and Critical Care Medicine, National Yang-Ming University School of Medicine, Taipei, Taiwan; 5 Department of Surgery, National Cheng Kung University Hospital, College of Medicine, National Cheng Kung University, No.138, Sheng Li Road, Tainan, 704 Taiwan; 6 Department of Pharmacology and Institute of Basic Medical Sciences, College of Medicine, National Cheng Kung University, No.1, University Road, Tainan, 70101 Taiwan

**Keywords:** Lung adenocarcinoma, DNA methylation array, Pyrosequencing, Risk score, Prognosis

## Abstract

**Background:**

The incidence of lung adenocarcinoma (LUAD) is increasing worldwide with different prognosis even in early-stage patients. We aimed to identify a prognostic panel with multiple DNA methylation biomarkers to predict survival in early-stage LUAD patients of different racial groups.

**Methods:**

The methylation array, pyrosequencing methylation assay, Cox regression and Kaplan-Meier analyses were conducted to build the risk score equations of selected probes in a training cohort of 69 Asian LUAD patients. The risk score model was verified in another cohort of 299 Caucasian LUAD patients in The Cancer Genome Atlas (TCGA) database.

**Results:**

We performed a Cox regression analysis, in which the regression coefficients were obtained for eight probes corresponding to eight genes (*AGTRL1*, *ALDH1A3, BDKRB1*, *CTSE*, *EFNA2*, *NFAM1*, *SEMA4A* and *TMEM129*). The risk score was derived from sum of each methylated probes multiplied by its corresponding coefficient. Patients with the risk score greater than the median value showed poorer overall survival compared with other patients (*p* = 0.007). Such a risk score significantly predicted patients showing poor survival in TCGA cohort (*p* = 0.036). A multivariate analysis was further performed to demonstrate that the eight-probe panel association with poor outcome in early-stage LUAD patients remained significant even after adjusting for different clinical variables including staging parameters (hazard ratio, 2.03; *p* = 0.039).

**Conclusions:**

We established a proof-of-concept prognostic panel consisting of eight-probe signature to predict survival of early-stage LUAD patients of Asian and Caucasian populations.

**Electronic supplementary material:**

The online version of this article (doi:10.1186/s12929-016-0276-x) contains supplementary material, which is available to authorized users.

## Background

Lung cancer is the leading cause of cancer-related deaths with an increasing incidence of lung adenocarcinoma (LUAD) subtype worldwide [1]. Prognosis may vary in patients with the same stage tumor because cancer is characterized by genetic, epigenetic, and phenotypic changes that result in a tremendous variability in clinical behavior [2, 3]. Therefore, the development of additional molecular markers for survival prediction of LUAD is required.

DNA methylation, which usually occurs in CpG dinucleotides, is a major epigenetic modification in mammalian genome [4–6]. High-throughput methylation arrays are now available to determine DNA methylation levels of thousands of CpG sites, simultaneously [7–9]. This technology enables large-scale DNA methylation analysis to identify informative DNA methylation biomarkers in lung cancer [7, 10–16]. Many reports have demonstrated that each cancer subtypes such as lung adenocarcinoma and squamous cell carcinoma has its own methylation signature [12, 13, 15, 16].

Therefore, in the current study we focus on the development of survival predictors in early-stage LUAD patients by performing genome-wide methylation analysis and pyrosequencing quantitative methylation assay to select eight DNA methylation probes in a training cohort of 69 patients recruited in Taipei Veterans General Hospital (TVGH). We also included certain clinical parameters that are known to affect prognosis [2, 3, 17–19] along with the selected eight-probe panel to the Cox regression analysis. The relevance of our finding has been validated in a cohort of 299 patients as part of The Cancer Genome Atlas (TCGA) project.

## Methods

### Patients and tissue samples

A total of 69 surgically resected LUAD patients in early stage (stages I and II) were recruited from Taipei Veterans General Hospital (TVGH), after obtaining appropriate institutional review board permission (#98-03-18A) and informed consent from the patients. These LUAD patients with checked clinical data and sufficient amount of DNA available for successful genome-wide methylation and pyrosequencing quantitative methylation assays were defined as a training cohort. A validation cohort of 299 LUAD patients with clinical follow-up data and methylation microarray data available from TCGA were collected. The mean follow-up period for training cohort was 82 months (range 9–157 months) and for validation cohort was 37 months (range 12–242 months). The end of the follow-up in TVGH was defined as January 2016 and TCGA as April 2015. Patients with clinicopathological characteristics are shown in Table 1.

**Table 1:** Characteristics of the lung adenocarcinoma patients included in the current study

Cohort	TVGH^a^ (%)	TCGA^a^ (%)
	*N* = 69 (100 %)	*N* = 299 (100 %)
Age		
< 65 year-old	25 (36.2 %)	124 (41.5 %)
≥ 65 year-old	44 (63.8 %)	166 (55.5 %)
Stage		
Stage IA	13 (18.8 %)	113 (37.8 %)
Stage IB	42 (60.9 %)	104 (34.8 %)
Stage IIA	4 (5.8 %)	32 (10.7 %)
Stage IIB	10 (14.5 %)	50 (16.7 %)
T stage		
Stage 1	16 (23.2 %)	122 (40.8 %)
Stage 2	51 (73.9 %)	159 (53.2 %)
Stage 3	2 (2.9 %)	18 (6.0 %)
N stage		
N0	56 (81.2 %)	236 (78.9 %)
≥ N1	12 (17.4 %)	57 (19.1 %)
M stage		
M0	69 (100 %)	299 (100 %)
≥ M1	0 (0.0 %)	0 (0.0 %)
Surgery		
Lobectomy	60 (87.0 %)	-^b^
Wedge resection	8 (11.6 %)	-^b^
Segmentectomy	1 (1.4 %)	-^b^
Chemotherapy		
No	51 (73.9 %)	-^b^
Yes	15 (21.7 %)	-^b^
TKI treatment		
No	62 (89.9 %)	-^b^
Yes	6 (8.7 %)	-^b^

^a^TVGH: Taipei Veterans General Hospital; TCGA: The Cancer Genome Atlas

^b^Information was not available for patients from TCGA

### Genomic DNA extraction and sodium bisulfite conversion

Genomic DNA from primary tumor tissue samples of 69 patients from TVGH were extracted using proteinase K digestion and phenol-chloroform extraction. A total of 1 μg genomic DNA was used for bisulfite conversion using the EpiTect Bisulfite kit (Qiagen, Duesseldorf, Germany) according to the manufacturer’s protocols.

### The genome-wide methylation analysis platform

The Illumina Infinium HumanMethylation27 BeadChip (27,578 CpG dinucleotides for 14,495 genes) was adapted for DNA methylation detection according to manufacturer’s manual. DNA methylation levels were reported as β-values by calculating the ratio of intensities between locus-specific methylated and unmethylated bead-bound probes. The β-value is a continuous variable, ranging from 0 (unmethylated) to 1 (fully methylated). The methylation array data can be viewed online under GEO accession number GSE83845.

### Pyrosequencing assay

To quantify cytosine methylation in individual CpG sites of candidate methylation probes identified by methylation array, bisulfite-converted DNA was analyzed using a pyrosequencing system (PyroMark Q24, Qiagen, Hilden, Germany). Specific pyrosequencing primer and PCR primer were designed for “target” CpG sites in the probes to be analyzed. Pyrosequencing was carried out in accordance with the manufacturer’s protocol (Qiagen). The target CpG sites were evaluated by converting the resulting pyrograms to numerical values for peak heights. Primer sequences are listed in Additional file 1: Table S1, and the genomic map of the detected CpG sites are shown in Additional file 1: Figure S1.

### Data processing and statistical analysis

Receiver operating characteristic (ROC) curve analysis was performed to determine the accuracy of the established CpG panel [area under the curve (AUC), sensitivity, and specificity]. The univariate and multivariate Cox regression analyses were conducted to explore the relationship between patient survival and several explanatory variables for defining the hazard ratio (HR) and confidence intervals (CI) of cancer death risk of variables using the Statistical Package for the Social Sciences version 17.0 (SPSS Inc., Headquarters Chicago, IL, USA). Overall survival curves were calculated according to the Kaplan-Meier method. *p* < 0.05 was considered statistically significant.

## Results

### Marker discovery in genome-scale DNA methylation dataset

**Fig. 1:**
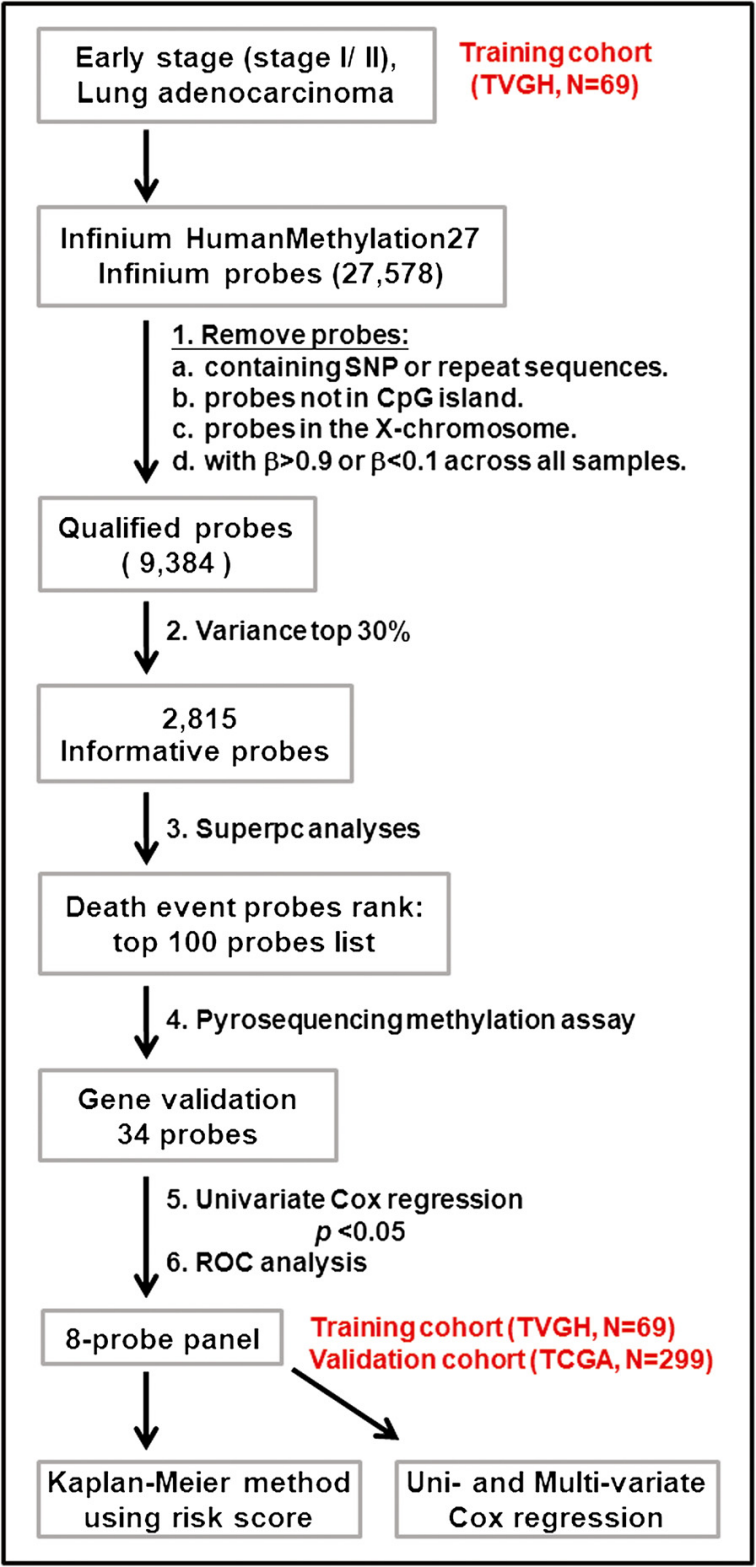
Flowchart of the probe selection and clinical validation procedures. Six steps were used to select the eight methylation gene probes from the methylation array in the training cohort of 69 LUAD patients from TVGH. The Kaplan-Meier survival analysis with the regression coefficients of eight probes was first performed to confirm the survival prediction of risk score calculation. The multivariate Cox regression was then performed to validate clinical performance of the eight-probe panel after adjusting for different clinical variables. The Kaplan-Meier survival analysis and multivariate Cox regression method were also performed in the validation cohort of 299 LUAD patients from TCGA database

**Fig. 2:**
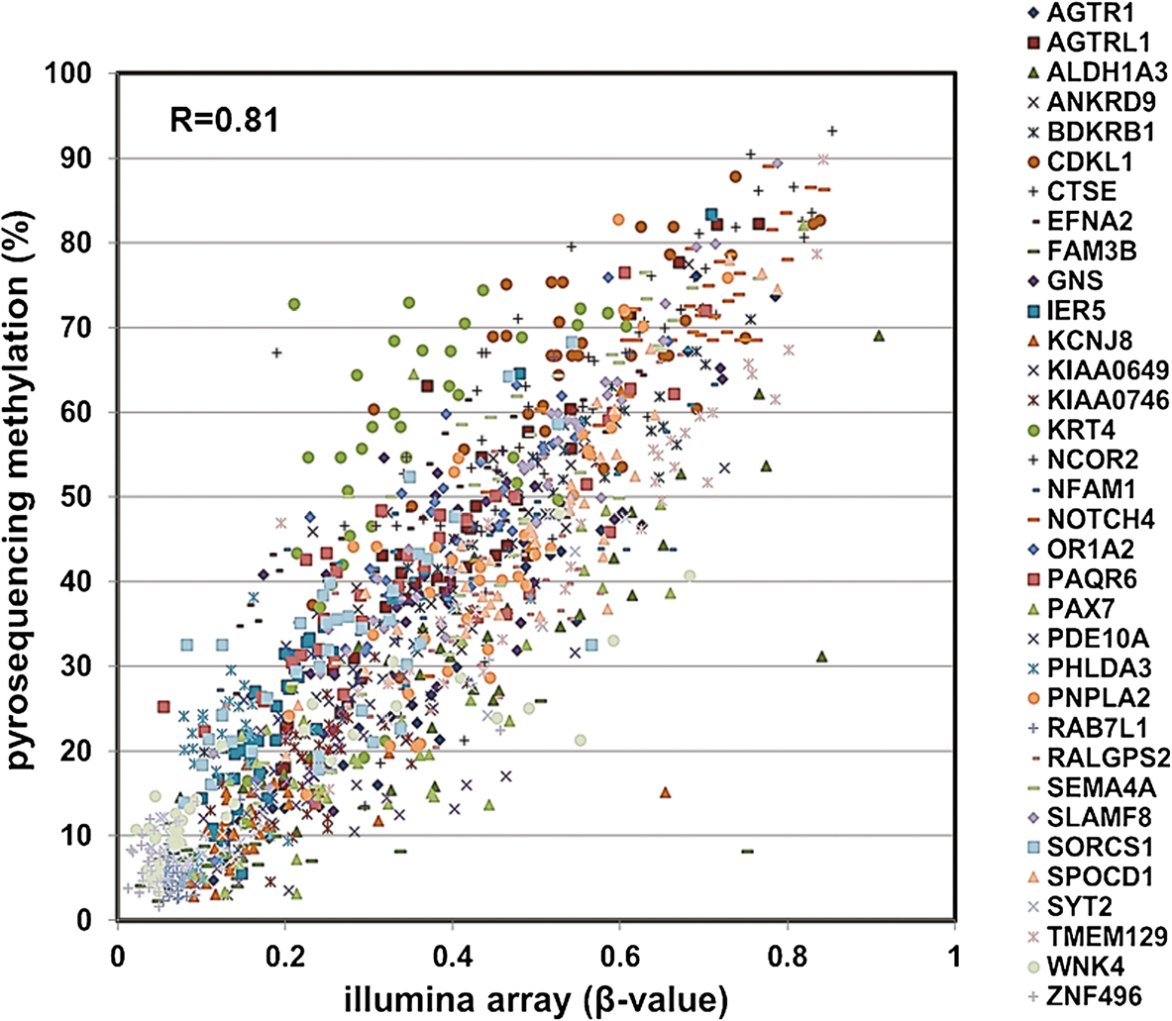
Correlation of methylation level between Illumina array and pyrosequencing method of top 34 methylated probes in early-stage LUAD patients. Dot-plot analyses show a high concordance of methylation level between pyrosequencing DNA methylation assay (Y-axis: %) and Illumina genome-wide methylation assay (X-axis: β value) and of 34 methylated probes. An average correlation coefficient was 0.81 (*R* = 0.81) among the probes

In the marker selection phase of this study, we collected surgically dissected tumors of 69 early-stage LUAD patients from TVGH to form a training cohort for genome-wide methylation array analysis using Illumina Infinium HumanMethylation27 BeadChip. Procedures were performed as described below and shown in Fig. 1. First, we obtained 9384 qualified probes after removing potentially problematic probes, probes containing SNP, repeat sequencing, probes not in CpG island, probes in the X-chromosome and the non-differential probes with β-value greater than 0.9 or less than 0.1 in all samples after methylation analysis. Second, we selected 2815 informative probes with large variance of β_tumor_ (top-30 % ranked) among the patients. Third, for each probe, a supervised principal components (Superpc) analysis [20] was applied to compare the survival distributions between patients with methylation levels above and below the mean level of all tumor tissues analyzed; 100 probes were chosen based on these tests. Fourth, since pyrosequencing assay is a highly sensitive method for detection of DNA methylation [12, 17, 21], we performed pyrosequencing methylation analyses. Of these probes, pyrosequencing was successfully designed and performed for 34 probes. A high concordance in quantifying the CpG methylation level was observed between DNA methylation array and pyrosequencing assay (Fig. 2). Fifth, eight specific candidate probes corresponding to eight genes showed significant correlation with survival by univariate Cox regression, including *Angiotensin II receptor-like 1* (*AGTRL1*), *Aldehyde dehydrogenase 1 family member A3* (*ALDH1A3*), *Bradykinin receptor B1* (*BDKRB1*), *Cathepsin E* (*CTSE*), *Ephrin A2* (*EFNA2*), *NFAT activating protein with ITAM motif 1* (*NFAM1*), *Semaphorin 4A* (*SEMA4A*), and *Transmembrane protein 129* (*TMEM129*) (Table 2). Sixth, we applied receiver operating characteristic (ROC) curve analysis to determine the diagnostic sensitivity and specificity of eight-probe panel in the validation cohort of TVGH patients. Finally, Kaplan-Meier method and multivariate Cox regression analyses were performed for the eight-probe panel in the TVGH patients and validated in 299 early-stage LUAD patients from TCGA datasets (as described below).

**Table 2:** Univariate Cox model for 34 probes in the training cohort of LUAD by pyrosequencing methylation assay

No.	probes ID^a^	gene symbol	*p* value^b^	No.	probes ID^a^	gene symbol	*p* value^b^
1	cg04878152	*AGTR1*	0.680	18	cg05973262	*NOTCH4*	0.126
2	cg25072179	***AGTRL1*** ^*c*^	0.001	19	cg16678925	*OR1A2*	0.164
3	cg27652350	***ALDH1A3*** ^*c*^	0.007	20	cg10046892	*PAQR6*	0.386
4	cg16787352	*ANKRD9*	0.223	21	cg11428724	*PAX7*	0.444
5	cg10528989	***BDKRB1*** ^*c*^	0.021	22	cg01431114	*PDE10A*	0.356
6	cg16077929	*CDKL1*	0.373	23	cg13645078	*PHLDA3*	0.877
7	cg21478437	***CTSE*** ^*c*^	0.007	24	cg24427660	*PNPLA2*	0.443
8	cg11885098	***EFNA2*** ^*c*^	0.002	25	cg09635067	*RAB7L1*	0.679
9	cg03158400	*FAM3B*	0.219	26	cg10559803	*RALGPS2*	0.175
10	cg00626466	*GNS*	0.384	27	cg15983538	***SEMA4A*** ^*c*^	0.030
11	cg13228642	*IER5*	0.275	28	cg04275881	*SLAMF8*	0.103
12	cg01226811	*KCNJ8*	0.564	29	cg16415058	*SORCS1*	0.265
13	cg17536532	*KIAA0649*	0.334	30	cg15789095	*SPOCD1*	0.811
14	cg10150813	*KIAA0746*	0.672	31	cg22594309	*SYT2*	0.944
15	cg12610744	*KRT4*	0.061	32	cg21505886	***TMEM129*** ^*c*^	0.004
16	cg22820108	*NCOR2*	0.546	33	cg08108311	*WNK4*	0.296
17	cg17568996	***NFAM1*** ^*c*^	0.002	34	cg01184522	*ZNF496*	0.560

^a^Probes ID is the CpG number of designated probe used in Illumina Human Methylation27 Bead Chip

^b^Univariate Cox regression

^c^Genes in bold font indicated statistical significance (*p* < 0.05) thus were selected for further analyses

### Sensitivity and specificity of the eight selected probes by ROC analysis

**Fig. 3:**
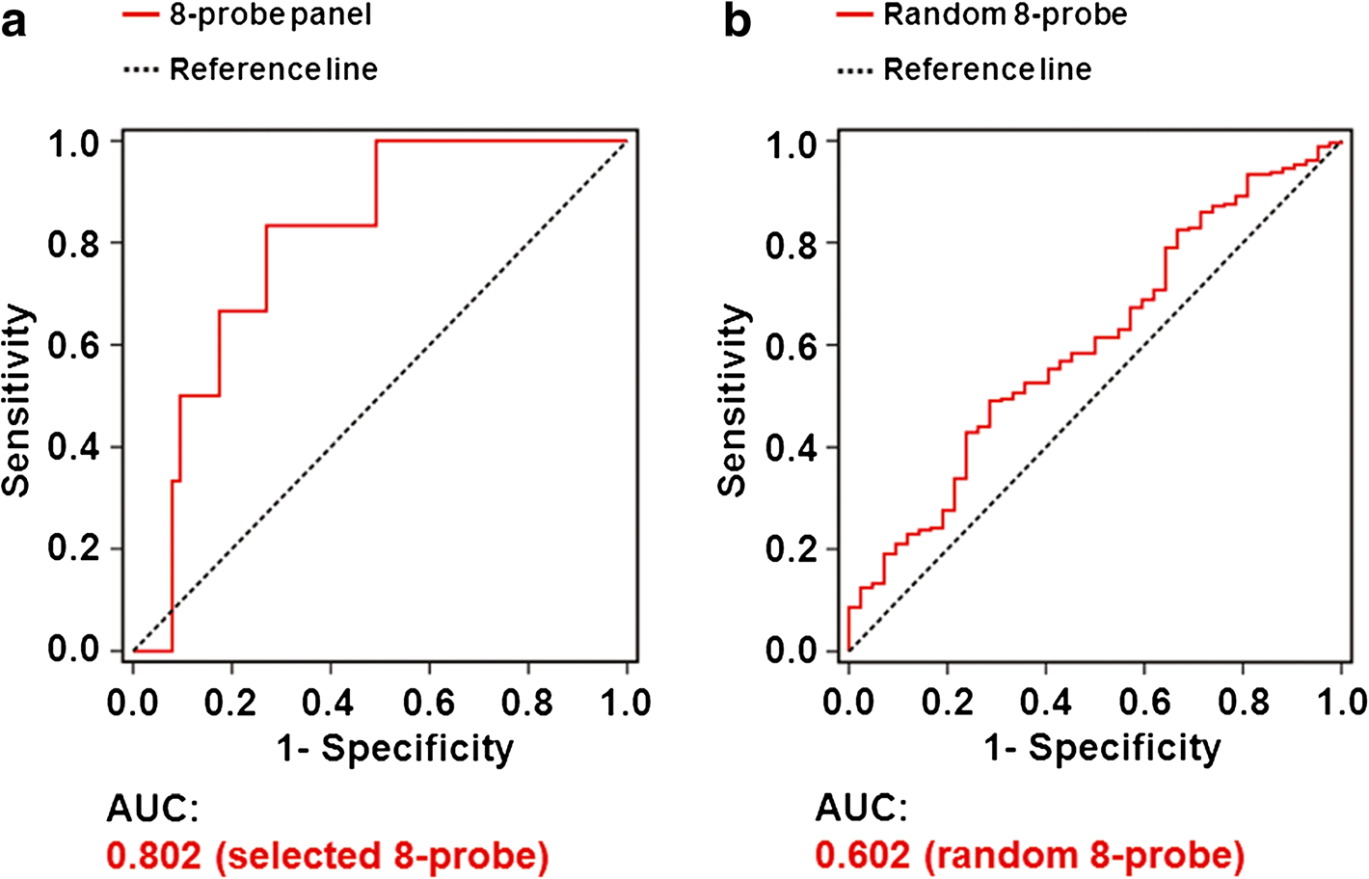
ROC curves of the prognostic predictor panel in the training cohort from TVGH. Sensitivity is indicated in the Y-axis, whereas 1 substrated by specificity (1-Specificity) is indicated in the X-axis. **a** The area under the curve (AUC) of ROC analysis for the eight selected probes panel. **b** The AUC of ROC analysis for the eight randomly selected genes

We examined the sensitivity and specificity of the eight selected probes by ROC curve analysis in the training cohort of 69 early-stage LUAD patients from TVGH. The area under the curve (AUC) of eight-probe together was 0.802 (Fig. 3a), indicating that the eight-probe signature showed good sensitivity and specificity in the ROC analysis. To assess the accuracy of the prognostic predictor panel, ROC curve analysis was performed on another randomly selected eight probes from the top 34 candidate probes. The AUC was 0.602 (Fig. 3b), suggesting a stronger prediction power of the specifically selected eight probes than the randomly selected probes. Thus, we defined this eight-probe signature as the prognostic predictor panel of early-stage LUAD.

### The risk score calculation and survival prediction of the eight-probe panel by Kaplan-Meier method

In the clinical validation phase, we first built the risk score for the eight selected methylation probes using the multivariate Cox regression analysis in the TVGH training cohort of 69 early-stage LUAD patients. These DNA methylation probe covariates were weighted by the regression coefficients to calculate the coefficient and hazard ratio for each patient. The risk score for each patient was derived from sum of methylation value of each probe multiplied by the corresponding coefficient, as following equation: risk score = *AGTRL1* methylation value × (-0.015) + *ALDH1A3* methylation value × (-0.023) + *BDKRB1* methylation value × (-0.034) + *CTSE* methylation value × (0.022) + *EFNA2* methylation value × (0.010) + *NFAM1* methylation value × (-0.017) + *SEMA4A* methylation value × (-0.012) + *TMEM129* methylation value × (-0.006). Example of risk score calculation for two patients is shown in Additional file 1: Figure S2.

**Fig. 4:**
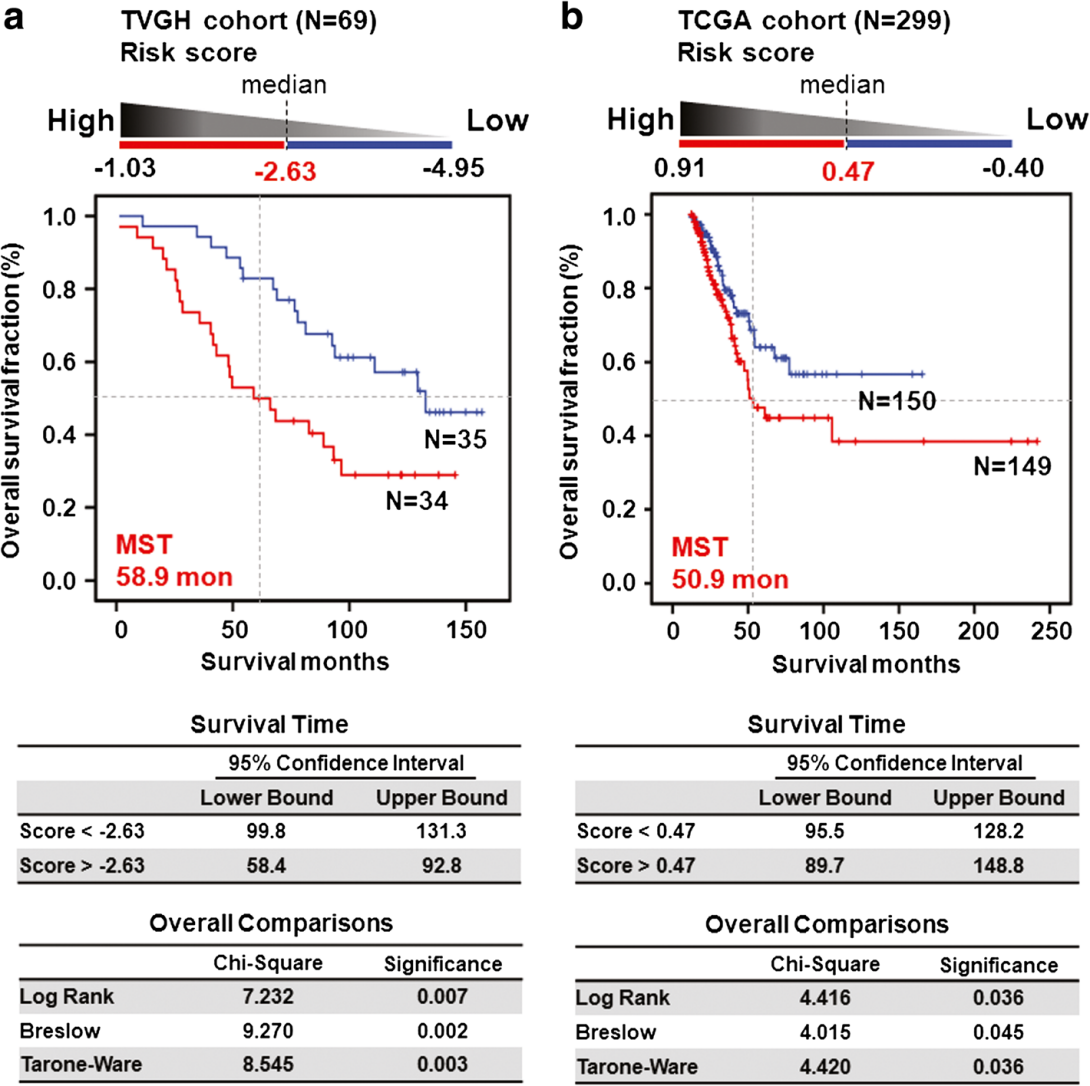
Survival risk score prediction based on the selected eight-probe in LUAD patients. **a** The risk score was used to classify 69 TVGH patients in the training cohort into two groups by median (as -2.63) (upper). The Kaplan-Meier overall survival analysis was performed to show the relative median survival time (MST) in two groups identified by the risk score calculation (middle). The 95 % confidence interval of survival time and *p* values of various methods are shown as indicated (lower). **b** The risk score, MST, and *p* values were analyzed in the validation cohort of 299 LUAD patients from TCGA database

Furthermore, we used the risk score calculation ranging from -1.03 to -4.95 to classify patients into two groups by median value of -2.63 in the TVGH training cohort of 69 early-stage LUAD patients (upper panel, Fig. 4a). The Kaplan-Meier overall survival analysis was performed to show the relative survival in each of the two groups identified by the risk score calculation (middle panel, Fig. 4a). Patients with high risk score indeed had a short median survival time (MST) of 58.9 months compared with other patients. The difference in the MST and 95 % confidence interval (CI) between the two groups was highly significant (lower panel, Fig. 4a). Therefore, the median risk score (as -2.63) was chosen as the cutoff value for survival prediction in the TVGH cohort.

We further applied our risk score model to determine whether our finding could be validated in another cohort of 299 early-stage LUAD patients whose follow-up data were available in TCGA project, and methylation level was also determined by the Infinium Methylation array. The risk score calculated with the median value (as 0.47) classified the 299 TCGA patients into two groups (upper panel, Fig. 4b). Such a calculation predicted a subset of patient with a high risk score showing poorer survival with MST of 50.9 months (middle panel, Fig. 4b) with statistical significance (lower panel, Fig. 4b). These results indicated that the prognostic predictor panel consisting of the selected eight-probe showed a strong prediction value in the TCGA validation cohort.

### Univariate and multivariate Cox regression analysis of the eight-probe panel

To determine whether the eight-probe panel is an independent variable associated with poor survival of early-stage LUAD patients, we performed the univariate and multivariate Cox regression model in both TVGH and TCGA cohorts. The univariate Cox regression analysis revealed that patients with risk score > median of the eight-probe panel, stage IIA, stage IIB, or lymph node metastases had poor outcome (*p* = 0.009, HR = 2.37, 95 % CI = 1.24–4.53 for risk score > median of the eight-probe panel; Table 3). Notably, multivariate Cox regression analysis showed that the eight-probe panel correlated with a relative risk of death of 2.03 (*p* = 0.039), even after adjusting for the tumor staging and metastasis status (Table 3), suggesting that the eight-probe panel was an independent risk factor of poor outcome.

**Table 3:** Univariate and multivariate Cox regression analyses of risk factors for cancer-related death in early-stage LUAD patients

	TVGH (*N* = 69)^a^				TCGA (*N* = 299)^a^			
Characteristics	Univariate analysis		Multivariate analysis^c^		Univariate analysis		Multivariate analysis^c^	
	HR (95 % CI)^b^	*p*-value^b^	HR (95 % CI)^b^	*p*-value^b^	HR (95 % CI)^b^	*p*-value^b^	HR (95 % CI)^b^	*p*-value^b^
Eight-probe panel								
Risk < Median	1.00		1.00		1.00		1.00	
Risk > Median	2.37 (1.24–4.53)	**0.009**	2.03 (1.04–3.98)	**0.039**	1.66 (1.03–2.66)	**0.038**	1.57 (0.96–2.57)	0.073
Gender								
Male	1.00		-		1.00		-	
Female	1.43 (0.71–2.88)	0.321	-	-	0.78 (0.49–1.26)	0.315	-	-
Stage								
Stage IA	1.00		1.00		1.00		1.00	
Stage IB	2.13 (0.73–6.16)	0.164	2.01 (0.69–5.86)	0.199	1.15 (0.63–2.11)	0.642	1.17 (0.63–2.16)	0.616
Stage IIA	8.76 (2.10–36.53)	**0.003**	5.79 (0.72–46.58)	0.099	2.17 (0.98–4.81)	0.057	0.85 (0.21–3.38)	0.817
Stage IIB	6.11 (1.81–20.64)	**0.003**	3.65 (0.70–18.92)	0.124	2.04 (1.07–3.91)	**0.031**	0.91 (0.26–3.15)	0.885
T stage								
Stage 1–2	1.00		-		1.00		-	
Stage 3–4	2.22 (0.53–9.28)	0.277	-	-	0.62 (0.15–2.53)	0.502	-	-
T stage								
Stage 1	1.00		-		1.00		-	
Stage 2	1.23 (0.56–2.68)	0.613	-		1.28 (0.77–2.12)	0.338	-	
Stage 3	2.59 (0.54–12.32)	0.232	-	-	0.72 (0.17–3.07)	0.657	-	-
N stage								
N0	1.00		1.00		1.00		1.00	
≥ N1	3.95 (1.88–8.30)	**0.001**	1.54 (0.35–6.91)	0.571	2.27 (1.38–3.73)	**0.001**	2.51 (0.74–8.43)	0.138
Chemotherapy								
No	1.00		-					
Yes	1.84 (0.92–3.69)	0.084	-	-				
TKI treatment								
No	1.00		-					
Yes	2.03 (0.79–5.26)	0.144	-	-				
Surgery								
Lobectomy	1.00		-					
Wedge resection	1.35 (0.53–3.45)	0.536	-					
Segmentectomy	2.98 (0.40–22.31)	0.288	-	-				

^a^TVGH: Taipei Veterans General Hospital; TCGA: The Cancer Genome Atlas

^b^CI, confidence interval; HR, hazard ratio. Bold values indicate statistical significance (*p* < 0.05)

^c^The variables without significant HR in the univariate analysis were not included in the multivariate analysis

To further define the prognostic effects of the eight-probe panel in early-stage LUAD patients, univariate and multivariate Cox regression analyses were performed in the TCGA validation cohort of 299 early-stage LUAD patients. Univariate Cox regression analysis revealed that patients with the risk score > median of the eight-probe panel had poor outcome, with a relative risk of death of 1.66 (*p* = 0.038) (Table 3). However, the eight-probe panel showed a borderline significance by the multivariate analysis in the TCGA cohort.

## Discussion

The incidence of LUAD is increasing worldwide [1]. Patients with the same stage of lung cancer may have different prognosis [22]. Development of prognostic markers is especially important in the patients with early-stage lung cancer, in whom clinical oncologists need selection factors to decide whether adjuvant therapy is necessary. In the present study, we develop a prognostic predictor panel for early-stage LUAD patients. This panel consists of eight DNA methylation probes corresponding to eight specific genes, including *AGTRL1, ALDH1A3, BDKRB1, CTSE, EFNA2, NFAM1, SEMA4A*, and *TMEM129*. The risk score calculated using the eight-probe panel served as an independent prognosis biomarker by Cox regression model and the multivariate analysis in our recruited patients. Therefore, the risk scores calculated from this eight-probe panel are valuable biomarkers for prognostic evaluation for early-stage LUAD patients to be tested in other cohorts.

Recently, Heller et al. identified a total of 12 genes that were differentially methylated in tumors compared with surrounding tissues in stage I, II or III Caucasian non-small cell lung cancer patients. Among the 12 genes, only the methylation patterns of *HOXA2* and *HOXA10* were independent prognostic factors in lung squamous cell carcinoma patients [15]. In addition, Esteller and the associates used methylation array to establish methylation profiles of stage I Caucasian non-small cell lung cancer and identified that methylation of two or more genes in *HIST1H4F*, *PCDHGB6*, *NPBWR1*, *ALX1*, and *HOXA9* correlated with an increased risk of cancer recurrence [16]. Interestingly, *HOXA9* promoter methylation was associated with high risk in stage I LUAD patients of two independently cohorts by another study [23]. To date, all studies that have been executed in an attempt to find markers for clinical use do not include patients from different racial groups. In our study, the prognostic predictor panel comprising eight DNA methylation biomarkers was an independent risk factor of poor outcome in Asian LUAD patients. We further applied our risk score model to determine whether our finding could be validated in another cohort of 299 early-stage LUAD patients whose follow-up data were available in TCGA project. The new coefficient and hazard ratio were defined according to the methylation value of the eight probes given in TCGA database of these patients. The Kaplan-Meier overall survival analysis showed that TCGA patients with risk score greater than median value had a shorter MST compared with other patients (Fig. 4b). However, the result of multivariate Cox regression was only close to significance in the Caucasian LUAD patients (Table 3). One of the limitations of the current TCGA study is that we are unable to acquire the data on treatment or surgery performed on the TCGA patients (Table 1). We believe that these results could be improved after including data from more patients when they are available in TCGA dataset or by validating in other cohorts of Caucasian LUAD patients.

The identification of the eight probes that can predict the clinical outcome in patients may reveal causes of the cancer development and tumorigenesis. For example, Angiotensin II receptor-like 1 (AGTRL1) and Bradykinin receptor B1 (BDKRB1) are G-protein-coupled receptors (GPCRs). GPCRs, which represent by far the largest family of cell-surface molecules involved in signal transduction, have recently emerged as crucial players in tumor growth and metastasis [24]. AGTRL1 is Apelin receptor. Apelin is an angiogenic factor secreted by tumor cells in order to promote the formation of new vessels necessary for tumor growth [25]. In addition, crosstalk between BDKRB1 and EGFR has been shown to maintain tumor growth in the breast cancer [26]. Aldehyde dehydrogenase 1 family, member A3 (ALDH1A3) is the retinoic acid biosynthesis enzyme, and plays a major role in the detoxification of aldehydes generated by alcohol metabolism and lipid peroxidation. Promoter hypermethylation of *ALDH1A3* has been reported to be a prognostic marker for lung cancer, gastric cancer, and invasive bladder cancer [27–30]. Cathepsin E (CTSE) prevents tumor growth and metastasis by catalyzing the proteolytic release of soluble trail from tumor cell surface [31]. Ephrin A2 (EFNA2), which belongs to ephrins family, regulates cell adhesion, motility, survival, proliferation, and differentiation. Semaphorins 4A (SEMA4A) suppresses endothelial cell migration and proliferation in vitro and angiogenesis in vivo mediated by vascular endothelial growth factor [32]. Further characterization of the probes validated in our panel could help to dissect the mechanism of LUAD tumorigenesis and progression.

The advantages of our prognostic predictor panel are as follows. First, the methylation level of the eight probes could be analyzed by DNA methylation array or pyrosequencing in patients. Second, the stepwise multivariate Cox regression analysis, in which the coefficients were obtained for the selected eight probes, could generate the risk score equations specifically for the cohort of patients to be tested. Third, any newly recruited patients could be assigned into risk groups once the risk score equations are determined. Therefore, the prognostic predictor panel could calculate the risk score not only in the Asian but also in the Caucasian LUAD patients. However, some technical limitations such as sample collection and preprocessing as well as experimental procedures of DNA methylation array or pyrosequencing assay need to be controlled to avoid batch effects. In addition, clinical variables such as adjuvant therapy and surgical methods may affect outcome prediction. Large-scale, multicenter and prospective studies are necessary to validate our risk score model in early-stage LUAD patients.

## Conclusions

Our study provides a proof-of-concept prognostic prediction panel consisting of eight methylated probes that are closely associated with survival in the early-stage LUAD patients. This prediction panel could be useful in stratifying patients according to the Cox-model and risk score before further treatment for early-stage LUAD patients who in dire need of intensive care.

## Abbreviations

AGTRL1, Angiotensin II receptor-like 1; ALDH1A3, Aldehyde dehydrogenase 1 family member A3; BDKRB1, Bradykinin receptor B1; CI, confidence intervals; CTSE, Cathepsin E; EFNA2, Ephrin A2; HR, hazard ratio; LUAD, lung adenocarcinoma; MST, median survival time; NFAM1, NFAT activating protein with ITAM motif 1; SEMA4A, Semaphorin 4A; Superpc, supervised principal components; TCGA, the cancer genome atlas; TMEM129, Transmembrane protein 129; TVGH, Taipei Veterans General Hospital

## Additional file

Additional file 1: Table S1. The primers used for pyrosequencing analysis^a^. **Figure S1.** The genomic maps of the selected genes and CpG sites in DNA methylation biomarker studies. cg_number is the CpG number of selected probes from methylation array. TSS: transcription start site. The black arrows (▼) indicate the detected CpG sites in Infinium array and the white arrows (△) indicate the sites in pyrosequencing. The nucleotides relative to TSS are shown. **Figure S2.** Risk score calculation and risk group assignment of two example patients. A Coefficient (coef) of genes and clinical variables were established by multivariate Cox regression model. A patient’s risk score was derived from sum of each probe methylation level multiplied by its corresponding coefficient. The equations used are as follows: Risk score = *AGTRL1* methylation value × (-0.015) + *ALDH1A3* methylation value × (-0.023) + *BDKRB1* methylation value × (-0.034) + *CTSE* methylation value × (0.022) + *EFNA2* methylation value × (0.010) + *NFAM1* methylation value × (-0.017) + *SEMA4A* methylation value × (-0.012) + *TMEM129* methylation value × (-0.006). B The risk score ranging from -1.03 to 4.95 was used to classify patients into two groups by the median value (as -2.63). Patient A with risk score of -1.0318 was assigned to the high risk group and patient B with -4.4262 was assigned to the low risk group. (DOCX 360 kb)
