# Supplementary material for: A prognostic predictor panel with DNA methylation biomarkers for early-stage lung adenocarcinoma in Asian and Caucasian populations
Source: J Biomed Sci. 2016 Aug 2;23:58. doi: 10.1186/s12929-016-0276-x (PMC4969679; doi:10.1186/s12929-016-0276-x)
Supplement: Additional file 1: Table S1. — The primers used for pyrosequencing analysisa. Figure S1. The genomic maps of the selected genes and CpG sites in DNA methylation biomarker studies. cg_number is the CpG number of selected probes from methylation array. TSS: transcription start site. The black arrows (▼) indicate the detected CpG sites in Infinium array and the white arrows (△) indicate the sites in pyrosequencing. The nucleotides relative to TSS are shown. Figure S2. Risk score calculation and risk group assignment of two example patients. A Coefficient (coef) of genes and clinical variables were established by multivariate Cox regression model. A patient’s risk score was derived from sum of each probe methylation level multiplied by its corresponding coefficient. The equations used are as follows: Risk score = AGTRL1 methylation value × (-0.015) + ALDH1A3 methylation value × (-0.023) + BDKRB1 methylation value × (-0.034) + CTSE methylation value × (0.022) + EFNA2 methylation value × (0.010) + NFAM1 methylation value × (-0.017) + SEMA4A methylation value × (-0.012) + TMEM129 methylation value × (-0.006). B The risk score ranging from -1.03 to 4.95 was used to classify patients into two groups by the median value (as -2.63). Patient A with risk score of -1.0318 was assigned to the high risk group and patient B with -4.4262 was assigned to the low risk group. (DOCX 360 kb) [file 12929_2016_276_MOESM1_ESM.docx]

**Supplementary Table 1.** The primers used for pyrosequencing analysis^a^

| **Gene** | **Primer name** | **Sequence ( 5'→3' )** | **PCR size (bp)** |
| --- | --- | --- | --- |
| *AGTRL1* | Forward | GGTGAAGGTGGGTGGTAGTTA | 103 |
|  | Biotin-Reverse | GGGACACCGCTGATCGTTTAAACCAAATTAACCCCTACTATACT |  |
|  | Sequencing | GTGGTAGTTAGAAAGGAG | -- ^b^ |
| *ALDH1A3* | Biotin-Forward | GGGACACCGCTGATCGTTTAAGTAGTAGAAAGAGGAGGAGTAGGA | 266 |
|  | Reverse | CCTCCAAAACCTCACCATCTTCTTTTAT |  |
|  | Sequencing | CCTCACCATCTTCTTTTATT | -- ^b^ |
| *BDKRB1* | Biotin-Forward | GGGACACCGCTGATCGTTTAAGAAGTTAATAGTGAAGGGGTTAAA | 170 |
|  | Reverse | AACTACCATCTCCCCCTTT |  |
|  | Sequencing | CCTACCCAAAACCTATATAT | -- ^b^ |
| *CTSE* | Forward | AGGTTTTGGAGTTATTAGTTATGTT | 230 |
|  | Biotin-Reverse | GGGACACCGCTGATCGTTTAACCTTTCCAAACAACCAAACATAT |  |
|  | Sequencing | AGTTATTAGTTATGTTTAGAGTAAA | -- ^b^ |
| *EFNA2* | Forward | AGGGTTGAGGTGTAGGGATTTAT | 84 |
|  | Biotin-Reverse | GGGACACCGCTGATCGTTTAACCTCCCTAAAACCCTCCTA |  |
|  | Sequencing | GGTGTAGGGATTTATTTTATT | -- ^b^ |
| *NFAM1* | Biotin-Forward | GGGACACCGCTGATCGTTTAAGGGTTAGGAGGTTAGATTGT | 262 |
|  | Reverse | TCTTCCTCCTTCTCTCTACTCT |  |
|  | Sequencing | CCAAAAACCTATCTCTAAAAATT | -- ^b^ |
| *SEMA4A* | Forward | GTTTTAGAGGTAGGGGATAGAAGG | 149 |
|  | Biotin-Reverse | GGGACACCGCTGATCGTTTACCCAAACCAAAATCTATCACAAC |  |
|  | Sequencing | GGTTTAGTTTGGTTGGA | -- ^b^ |
| *TMEM129* | Biotin-Forward | GGGACACCGCTGATCGTTTAAGGGAAGTTTAGAGTTGAGTAAT | 279 |
|  | Reverse | CAAAACCCTCCCCAACTTTAC |  |
|  | Sequencing | ATTCTCTCTAACAACCTTT | -- ^b^ |

^a^ Pyrosequencing was performed for the eight methylation probes identified by methylation array. Pyrosequencing-PCR was amplified by forward and reverse primers with one labeled with biotin to facilitate further purification. Pyrosequencing-sequencing primer was used to generate the quantitative methylation level of fragment generated by Pyrosequencing-PCR.

^b^ --: Not applicable.

**Supplementary Figures**


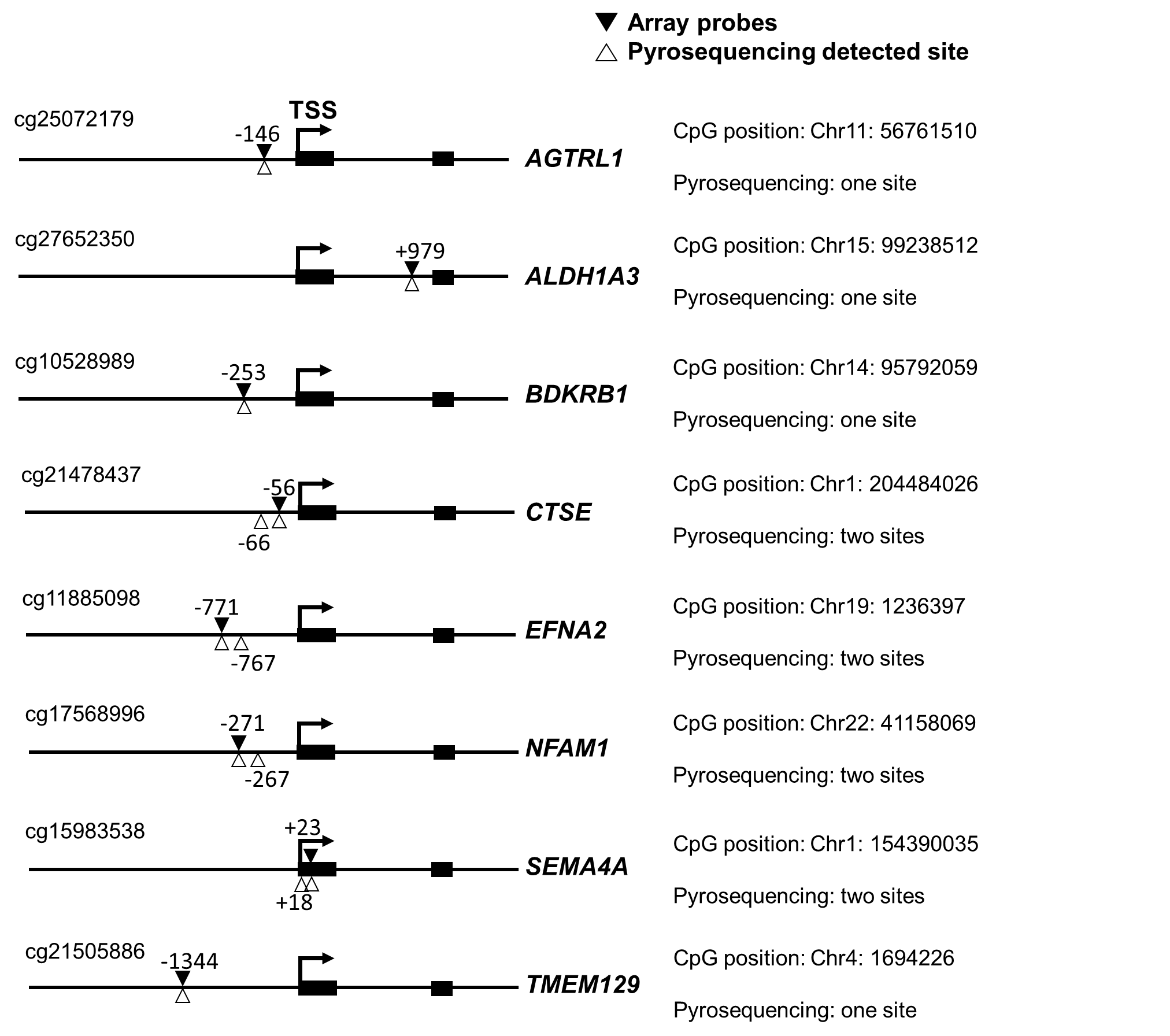


**Supplementary Figure 1.** The genomic maps of the selected genes and CpG sites in DNA methylation biomarker studies. cg_number is the CpG number of selected probes from methylation array. TSS: transcription start site. The black arrows (⯆) indicate the detected CpG sites in Infinium array and the white arrows (△) indicate the sites in pyrosequencing. The nucleotides relative to TSS are shown.

**
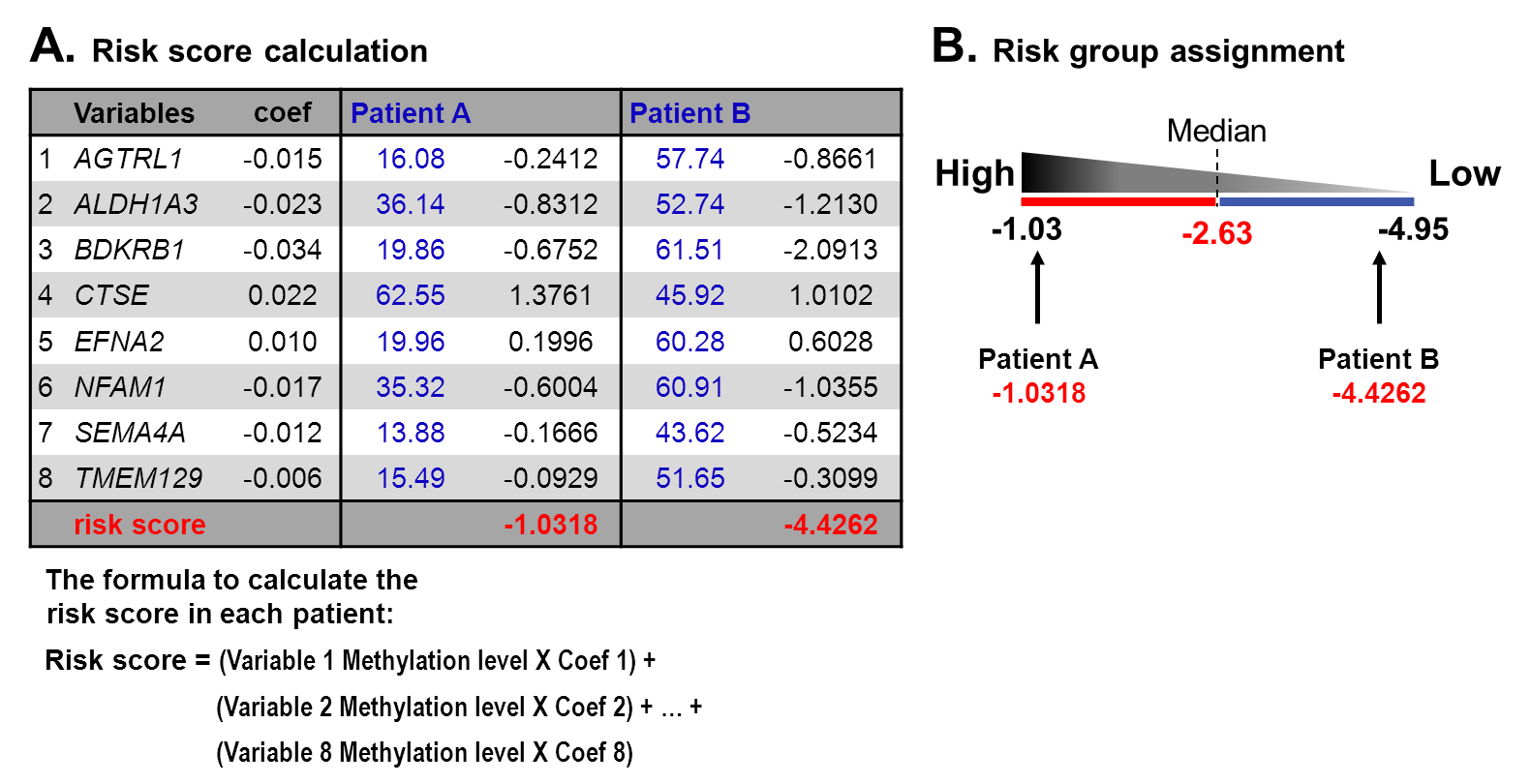
**

**Supplementary Figure 2.** Risk score calculation and risk group assignment of two example patients. **A** Coefficient (coef) of genes and clinical variables were established by multivariate Cox regression model. A patient’s risk score was derived from sum of each probe methylation level multiplied by its corresponding coefficient. The equations used are as follows: Risk score = *AGTRL1* methylation value × (-0.015) + *ALDH1A3* methylation value × (-0.023) + *BDKRB1* methylation value × (-0.034) + *CTSE* methylation value × (0.022) + *EFNA2* methylation value × (0.010) + *NFAM1* methylation value × (-0.017) + *SEMA4A* methylation value × (-0.012) + *TMEM129* methylation value × (-0.006). **B** The risk score ranging from -1.03 to 4.95 was used to classify patients into two groups by the median value (as -2.63). Patient A with risk score of -1.0318 was assigned to the high risk group and patient B with -4.4262 was assigned to the low risk group.
